# CBC3T-3: a novel patient-derived cisplatin-resistant distal cholangiocarcinoma cell line harboring multiple *TP53* missense mutations

**DOI:** 10.1007/s13577-026-01369-1

**Published:** 2026-03-31

**Authors:** Jiahui Xi, Mingzhen Bai, Ruyang Zhong, Chongfei Huang, Ruoshui An, Long Gao, Haidong Ma, Liang Tian, Jinyu Zhao, Ningzu Jiang, Xiang He, Leiqing Wang, Zihe Dong, Ping Yue, Yanyan Lin, Zhongtian Bai, Wenbo Meng

**Affiliations:** 1 The First School of Clinical Medicine, Lanzhou University, Lanzhou, 730000 China; 2 Department of General Surgery, The First Hospital of Lanzhou University, Lanzhou, 730000 Gansu China; 3 Key Laboratory of Biotherapy and Regenerative Medicine, Gansu Province, Lanzhou, 730000 China; 4 Department of Hepatobiliary Pancreatic Tumor Center, Chongqing University Cancer Hospital, Chongqing, China

**Keywords:** Distal cholangiocarcinoma, Cell line, Cisplatin, *TP53* missense mutation, Drug resistance

## Abstract

Distal cholangiocarcinoma (dCCA) is a malignant tumor characterized by a challenging diagnosis, high invasiveness, and extremely poor prognosis. Research on dCCA is limited by the scarcity of reliable patient-derived preclinical tumor models. This study established a novel human distal cholangiocarcinoma cell line, CBC3T-3, and systematically characterized its biological properties, genomic features, and potential for clinical application. This cell line was extracted from postoperative distal cholangiocarcinoma tumor from a 54-year-old male patient. It was stably passaged (> 50 generations) through primary culture and condition optimization, preserving the same pathology as that of the primary tumor. Whole-exome sequencing (WES) confirmed somatic mutations, tumor mutation burden, single-sample clonal structure, driver genes, and drug resistance genes in CBC3T-3 cells, revealing their genomic characteristics. Functional assays demonstrated that CBC3T-3 cells exhibit strong capabilities for proliferation, migration, and invasion in vitro. In a subcutaneous xenograft model in immunodeficient mice, palpable tumor nodules developed within 4 weeks, reflecting the clinical characteristics of rapidly progressive disease. Drug sensitivity analysis revealed that, compared with TFK-1 cells, CBC3T-3 cells presented significantly greater responses to paclitaxel, gemcitabine, and oxaliplatin but relatively poor responses to 5-FU and cisplatin. The integration of drug resistance gene findings from WES suggests that *TP53* missense mutations may mediate primary resistance to cisplatin. The establishment of the CBC3T-3 cell line enhances the research toolkit for dCCA. Its genomic characteristics and functional plasticity provide a reliable preclinical tumor model for developing precision therapies and investigating drug resistance mechanisms.

**Supplementary Information:**

The online version contains supplementary material available at 10.1007/s13577-026-01369-1.

## Introduction

Cholangiocarcinoma (CCA) is a cohort of highly heterogeneous malignancies that originate from the biliary tract. It constitutes approximately 3% of all gastrointestinal cancers, and the highest incidence rates are noted in Asia [1, 2]. Although CCA receives less attention than other malignancies do, its incidence rate is increasing [3]. CCAs can be categorized according to their anatomical site into intrahepatic cholangiocarcinoma, hilar cholangiocarcinoma, and distal cholangiocarcinoma (dCCA). Each anatomical subtype presents unique genetic, pathological, and clinical features [4, 5]. dCCA is a malignant tumor that originates at the distal end of the common bile duct and accounts for approximately 20–30% of all bile duct cancers [6]. Owing to its concealed anatomical location and atypical early symptoms, the majority of patients are diagnosed at the late stage. The surgical resection rate is less than 30%, and the 5-year post-operative survival rate is approximately 7–20% [7]. Although breakthroughs in targeted therapy and immunotherapy have been made in various solid tumors in recent years, effective systemic treatment options for dCCA remain extremely limited, and its significant heterogeneity poses challenges for diagnosis and treatment.

The significance of preclinical tumor models (cell lines, xenografts, and organoids) in precision medicine continues to grow, providing a vital platform for investigating tumor mechanisms and testing novel drug development [8, 9]. Patient-derived dCCA cell lines can retain the genetic variations, transcriptomic features and drug sensitivity of the primary tumor, providing an efficient platform for in vitro studies on tumor pathogenesis and high-throughput drug screening [10]. The genomic heterogeneity of dCCA is manifested in the differences in gene mutation profiles among different patients and in different tumor regions of the same patient. This heterogeneity affects the biological behavior of the tumor and its response to treatment [11, 12]. Therefore, establishing novel patient-derived dCCA cell lines is highly important for elucidating the genomic characteristics of dCCA, screening potential therapeutic targets, and developing individualized treatment strategies.

On the basis of the above background, this study successfully established and characterized a novel patient-derived dCCA cell line, CBC3T-3. By integrating primary cell culture techniques, whole-exome sequencing (WES) and functional experiments, we systematically evaluated the genomic characteristics, in vitro and in vivo growth features and drug response patterns of this cell line and verified its consistency with clinical samples.

## Materials and methods

### Patient Information

**Fig. 1:**
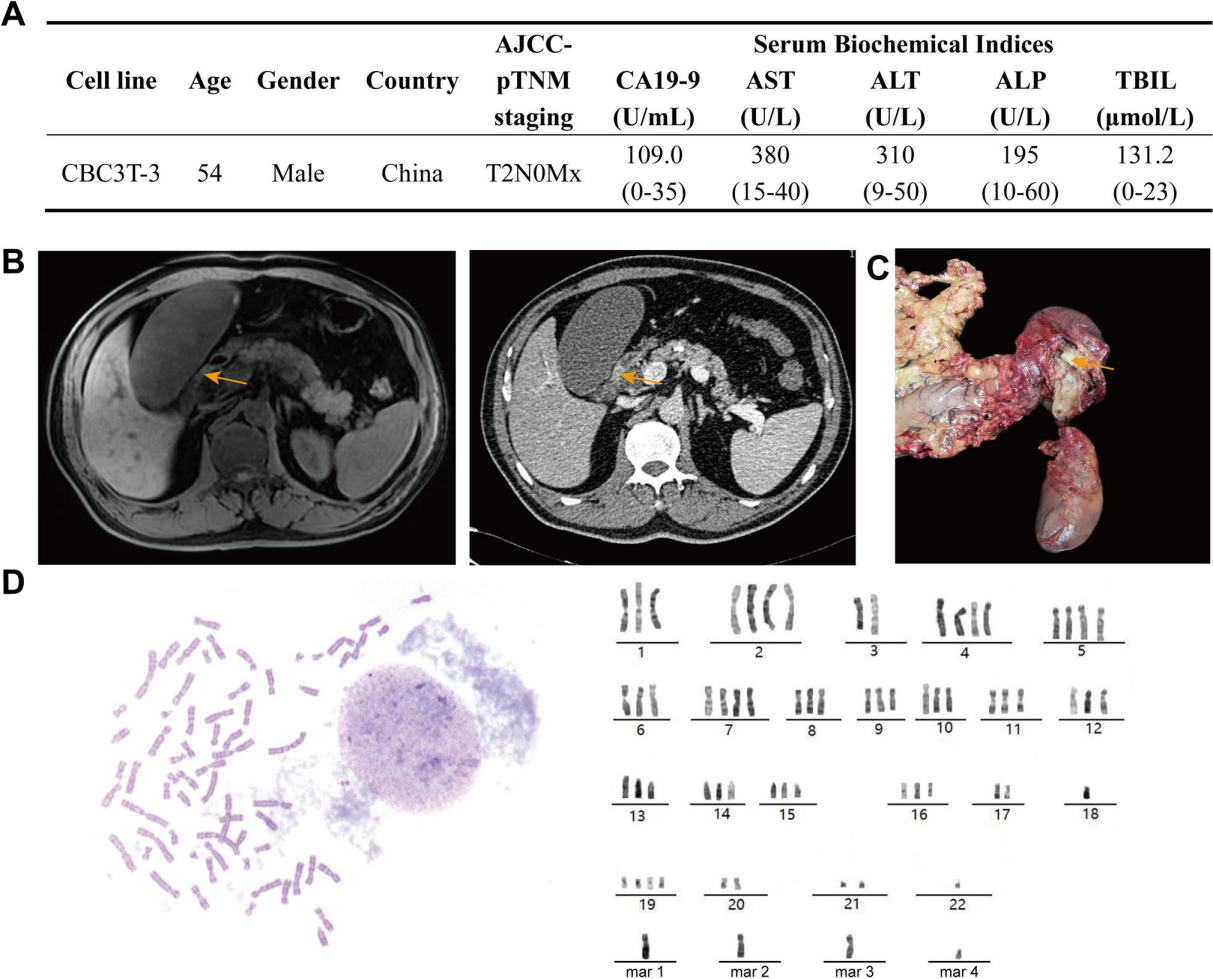
Clinical characteristics of the patient and identification of CBC3T-3 cells. **A** Clinical data of the patient. **B** Preoperative MRI and CT findings suggestive of distal cholangiocarcinoma. **C** Photograph of the surgical specimen obtained after pancreaticoduodenectomy, showing a white, firm tissue mass protruding into the lumen within the pancreatic segment of the common bile duct. **D** Chromosome karyotype analysis of CBC3T-3 cells

A 54-year-old male patient was admitted to the Department of General Surgery of The First Hospital of Lanzhou University in 2022. The main complaints were abdominal pain, distension, jaundice (manifestation of skin and sclera yellowing) and pruritus (skin itching) for more than 1 month. Laboratory tests revealed a significant elevation in the serum tumor marker CA19-9*,* and liver function tests indicated obstructive jaundice (Fig. 1A). Imaging studies (abdominal MRI and CT) revealed a space-occupying lesion in the distal common bile duct, which was highly suspicious for malignancy (Fig. 1B). The patient had a 10-year history of type 2 diabetes mellitus and had previously undergone bladder tumor resection. There was no relevant family history of genetic diseases. After multidisciplinary consultation, the patient underwent pancreaticoduodenectomy. During surgery, white, firm tumor tissue was observed at the distal extremity of the common bile duct (Fig. 1C). Postoperative pathological examination confirmed moderately to poorly differentiated CCA (AJCC-pTNM staging: T2N0Mx). The histological features were characterized by tumor cells arranged in cord-like or glandular patterns infiltrating within a desmoplastic collagenous stroma. The tumor cells exhibited cuboidal to columnar morphology with significant nuclear atypia and a high nuclear‒cytoplasmic ratio. Pathological mitotic figures and perineural invasion were observed. The immunohistochemical staining results were as follows: *CK19* (+), *TP53* (+), and the *Ki-67* proliferation index was 30–40%. Postoperative adjuvant chemotherapy with gemcitabine plus capecitabine was administered, and the patient recovered well. This study was approved by the Medical Ethics Committee of The First Hospital of Lanzhou University (Approval No. LDYYLL2025-954), and written informed consent was obtained from the patients.

### CBC3T-3 cell line establishment

Fresh sterile primary CCA tissue obtained during surgery was immediately placed in precooled DMEM/F-12 medium (Gibco, USA) containing 10% penicillin‒streptomycin dual antibiotics to maintain tissue viability. The tissue was rinsed three times with PBS containing 10% penicillin‒streptomycin dual antibiotics. Using sterile forceps, the fibrous capsule, adipose tissue, and visible blood vessels were carefully removed. The tissue was then minced into small pieces of 1–2 mm^3^ and transferred into DMEM/F-12 medium containing 200 U of collagenase II for enzymatic digestion for 10 min to obtain a single-cell suspension. Single-cell suspensions were seeded evenly into 6-well plates. After 48 h, the culture medium was replaced, and the cell growth status was observed. Fibroblasts were removed via mechanical scraping. When the cell confluence reached approximately 80%, the tumor cells were further isolated and purified via differential digestion.

### Cell culture

The human extrahepatic CCA cell line TFK-1 was procured from the National Biomedical Experimental Cell Resources Bank (Beijing, China). For cultivation, the cells were grown in RPMI 1640 medium (BI, Israel) containing 10% fetal bovine serum (ABW, USA) and 1% penicillin‒streptomycin solution (BI, Israel). The cells were incubated under standard conditions at 37 °C in a humidified incubator with 5% CO2.

### Short tandem repeat (STR) analysis

STR profiling was performed by the China Center for Type Culture Collection (CCTCC; Wuhan, China). In accordance with the ANSI/ATCC ASN-0002–2021 standard, a minimum of 13 STR loci were required for cell identity verification. To genotype CBC3T-3, 21 allele-specific STRs, such as CSF1PO, D5S818, D19S433, etc., were used in the STR multiplex reaction. The STR amplifications were resolved on an Applied Biosystems 3730XL Genetic Analyzer. The resulting STR profile was then mapped against international databases such as ATCC, DSMZ, and CELLOSAURUS to evaluate the degree of matching of this cell line with known STR profiles.

### Chromosome karyotype analysis

The cells were grown until the logarithmic growth phase and then incubated with colchicine at a final concentration of 0.2 μg/mL for 2 h to inhibit spindle formation and induce chromosome condensation. After treatment, the cells were harvested and lysed in 0.075 M KCl solution for hypotonic shock, which induced swelling for the dispersion of chromosomes. The cells were then fixed with freshly prepared methanol–glacial acetic acid fixative (3:1, v/v) to preserve the cellular morphology and remove residual hypotonic solution. The fixed cell suspension was dropped onto clean glass slides and dried at 70 °C for 2 h. Chromosomes were stained via Giemsa staining, observed under an optical microscope, and imaged for karyotype analysis.

### Cell growth curve

The 96-well plates were seeded with CBT3T-3 cells at a density of 5 × 10^3^ cells per well. At 6 h, and on days 1, 2, 3, and 4 after seeding, CCK-8 reagent (APExBio, USA) was added to the cells, followed by incubation in the dark for 2 h. The optical density (OD) was then measured at a wavelength of 450 nm. The data were statistically analyzed by using GraphPad Prism 8.0 software (GraphPad Software, USA), and a cell growth curve was plotted to evaluate the proliferation kinetics.

### Cell migration and invasion

Transwell inserts (8 μm pore size, Corning, USA) were precoated with 50 μL of Matrigel matrix (R&D Systems, USA), which was diluted at a 1:8 ratio with serum-free medium for 30 min at 37 °C for polymerization. After the uncoated medium was removed, 200 μL of a serum-free cell suspension containing 8 × 10^4^ cells was added to the upper chamber, while the lower chamber was filled with 700 μL of medium supplemented with 20% fetal bovine serum (Cell-Box, China) to establish a chemotaxis model. The system was cultured at 37 °C under 5% CO₂ for 48 h, and the cell status was monitored every 12 h. The culture was completed after the set time, after which the cells were fixed in 4% paraformaldehyde for 30 min and stained with 0.1% crystal violet for another 30 min. Images of three arbitrarily selected nonoverlapping fields were recorded via an inverted microscope. The invasive area was quantified via threshold analysis via ImageJ software, and we performed a statistical analysis via GraphPad Prism 8.0.

### Transmission electron microscopy

The cells were fixed with electron microscopy fixative (Servicebio, G1102, China) for 2–4 h, followed by three washes with 0.1 mol/L phosphate buffer. The samples embedded in 1% agarose were subjected to postfixation with 1% osmium tetroxide in 0.1 mol/L phosphate buffer. The fixation process lasted for 2 h at room temperature in the dark, after which the samples were thoroughly rinsed three times with the same buffer. After dehydration through a graded ethanol series, the samples were infiltrated with resin and polymerized. Ultrathin sections were obtained via a Leica UC7 (Germany) ultramicrotome, which were then subjected to staining with 2% uranyl acetate in saturated alcohol solution for 8 min in the dark and subsequently dried overnight. Final observation and imaging were conducted using a HITACHI HT7700 (Japan) transmission electron microscope at 80 kV.

### Scanning electron microscopy

The cells were fixed with electron microscopy fixative (Servicebio, G1102, China) for 2 h and then rinsed 3 times with 0.1 mol/L phosphate buffer. The samples were dehydrated via a stepwise ethanol gradient and dried via a cold dryer. A small amount of conductive glue was applied to the sample stage, and high-resolution cold-field emission scanning electron microscopy (HITACHI, SU8100, Japan) was used for image observation and acquisition.

### Drug sensitivity analysis

The 96-well plates were seeded with CBT3T-3 cells or TFK-1 cells at a density of 5 × 10^3^ cells per well. After 24 h of culture, different concentrations of gemcitabine, oxaliplatin, cisplatin, fluorouracil or paclitaxel were added for treatment. After 48 h of drug intervention, the original culture medium was discarded, and 200 μL of CCK-8 reagent was added to each well. The plates were incubated at 37 °C for another 2 h. The absorbance values of each well were detected at a wavelength of 450 nm via a microplate reader (BioTek Synergy H1, USA). The experimental data were statistically analyzed and graphed via GraphPad Prism 8.0 software.

### Cell line-derived xenografts

Four-week-old NOD-SCID mice were inoculated subcutaneously in the dorsal flank with 5 × 10⁶ CBC3T-3 cells in a 100 μL 1:1 PBS–Matrigel suspension. The animals were monitored every 4 days post-injection. Once the tumors developed, the longest (L) and shortest (W) diameters were measured via a Vernier caliper, and the tumor volume was calculated. At 10 weeks post-injection, the mice were euthanized, the subcutaneous tumors were excised for imaging and weighing, and portions of the tumor tissue were fixed for hematoxylin–eosin (HE) and immunohistochemical staining. The remaining tissues were stored at – 80 °C.

### H&E and immunohistochemical staining

For immunohistochemistry, formalin-fixed, paraffin-embedded tissues were sectioned and processed through deparaffinization, rehydration, antigen retrieval, and serum blocking. The sections were then incubated overnight at 4 °C with primary antibodies against Ki-67 (1:500, GB111499, Servicebio), TP53 (1:1000, GB12626, Servicebio), and CK19 (1:500, GB11197, Servicebio). This was followed by washing with PBS, incubation with species-matched secondary antibodies, and DAB chromogenic development. Hematoxylin was used for nuclear counterstaining, and the stained sections were visualized under a Nikon (Japan) microscope.

### WES

Libraries for exome sequencing were constructed from genomic DNA isolated from the patient's adjacent normal tissue and CBC3T-3 cells. The libraries were subjected to exome capture via the Agilent SureSelect Human All Exon V6 system (Novogene, Beijing, China) and sequenced on an Illumina NovaSeq 6000 platform. The generated raw data were aligned via BWA and interrogated via the GATK pipeline for variant calling. The spectrum of final annotated variants included single nucleotide polymorphisms (SNPs), insertions and deletions (InDels), and copy number variations (CNVs).

### Statistical analysis

The experiments in this research were performed in three biological replicates (n = 3). An unpaired two-tailed Student's t test was used to assess the significant differences between the compared groups. All the statistical analyses were performed via GraphPad Prism 8, and *P* < 0.05 was considered statistically significant.

## Results

### Construction and identification of CBC3T-3 cells

We succeeded in establishing and characterizing a new cell line from patient primary tumor tissue, named CBC3T-3. The initial isolation and purification of the cell line took approximately 3 months, and the development of a stable cell line (passaged over 50 generations) was completed in approximately 1 year. STR analysis confirmed the absence of human cell cross-contamination in CBC3T-3 cells. CBC3T-3 exhibited a unique STR profile that was highly concordant with the primary tumor but distinct from all database records, indicating a novel origin (Table 1). The bold values in Table 1 denote loci where allele differences were observed between the established cell line and the original tumor tissue. Specifically, the loss of heterozygosity at D18S51 and D6S1043 reflects genomic instability and clonal selection during in vitro culture. The loss of the Y chromosome identified in the CBC3T-3 cell line represents a hallmark of tumor genomic instability. FISH analysis using X and Y chromosome-specific probes confirmed the presence of X chromosome material and complete absence of the Y chromosome (Fig. S1). This phenomenon may partially underlie the epidemiological association between male sex and an increased incidence of certain cancers [13]. Additionally, the cells displayed a highly rearranged near-triploid karyotype (3n = 67) with complex numerical and structural chromosomal aberrations (Fig. 1D). No intact or cytogenetically identifiable Y chromosome was observed in the karyotype analysis, consistent with STR and FISH results. Therefore, all unidentifiable chromosomal elements were designated as marker chromosomes (mar) according to International System for Human Cytogenomic Nomenclature 2020 guidelines.

**Table 1:** The alleles of 21 loci in CBC3T-3 cell and tumor tissue

Marker	CBC3T-3 cell	Tumor tissue
D19S433	14, 14.2	14, 14.2
D5S818	10, 11	10, 11
D21S11	30	30
D18S51	**14, 14**	**12, 14**
D6S1043	**12, 12**	**12, 19**
AMEL	**X, X**	**X, Y**
D3S1358	15, 16	15, 16
D13S317	8, 8	8, 8
D7S820	11, 11	11, 11
D16S539	10, 11	10, 11
CSF1PO	10, 13	10, 13
Penta D	12, 12	12, 12
D2S441	11, 12	11, 12
vWA	16, 18	16, 18
D8S1179	12, 13	12, 13
TPOX	8, 11	8, 11
Penta E	11, 15	11, 15
TH01	9, 9	9, 9
D12S391	19, 21	19, 21
D2S1338	18, 23	18, 23
FGA	19, 25	19, 25

### Morphological characteristics of CBC3T-3 cells

**Fig. 2:**
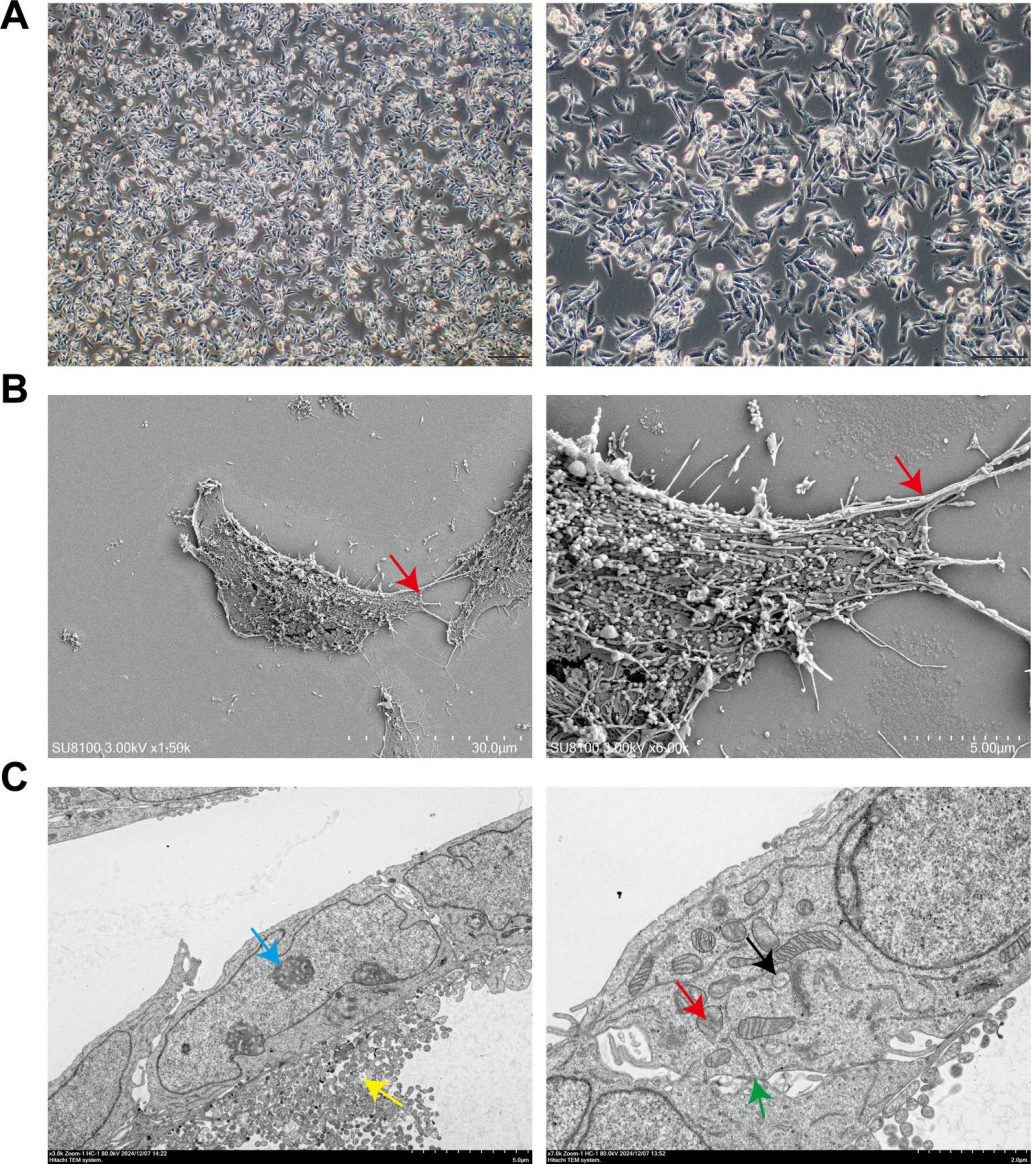
Morphological characteristics of CBC3T-3 cells. **A** Morphology of CBC3T-3 cells under light microscopy at 40 × and 100 × magnification. **B** Ultrastructural features observed by scanning electron microscopy. Red arrows: tight junctions. **C** Ultrastructural features observed by transmission electron microscopy. Yellow arrows: secretory granules; blue arrows: heterochromatin aggregation; red arrows: swollen mitochondria with disrupted cristae; black arrows: mitochondrial vacuolation; green arrows: desmosomes

Under light microscopy, CBC3T-3 cells exhibited adherent colony growth, with a morphology predominantly composed of spindle and polygonal shapes. The cells displayed large nuclei, a high nuclear‒cytoplasmic ratio, and visible cytoplasmic vacuoles (Fig. 2A). Scanning electron microscopy revealed abundant microvilli, pseudopodia, and irregular protrusions on the cell surface, along with tight junctions and intercellular bridges between adjacent cells (Fig. 2B). Transmission electron microscopy further revealed irregular nuclear contours, condensed heterochromatin, and a significantly increased nuclear‒cytoplasmic ratio. The cytoplasm contained numerous mitochondria, some of which exhibited swelling, disrupted cristae, or vacuolar degeneration. The rough endoplasmic reticulum and Golgi apparatus were relatively sparse, while the number of secretory granules increased. Desmosome structures were also observed (Fig. 2C).

### Genomic characteristics of CBC3T-3 cells

**Fig. 3:**
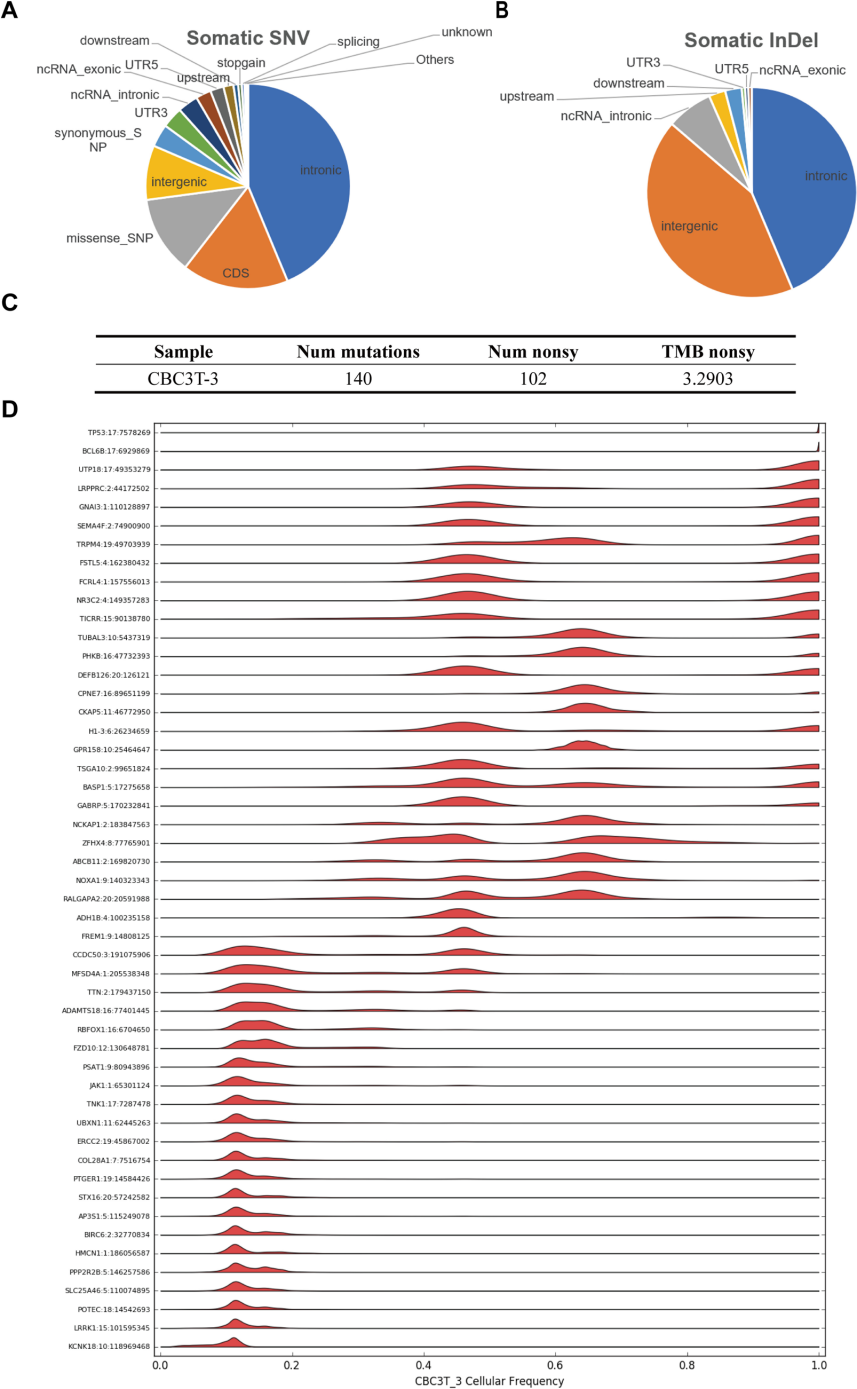
Genomic characteristics of CBC3T-3 cells.** A** Distribution of somatic SNVs across different genomic regions. **B** Distribution of somatic InDels across different genomic regions. **C** TMB in CBC3T-3 cells. **D** Single-sample clonal structure analysis showing 50 randomly selected mutations. The x-axis represents the mutant cell frequency, and the y-axis indicates the corresponding gene. The mutation frequency is color-coded in red; values closer to 1 indicate clonal mutations, whereas lower values suggest subclonal mutations

WES is a pivotal genomic technique widely used in genetic disease diagnosis, cancer research, and drug development [14]. By performing WES on tumor cells and matched normal tissues, it enables systematic identification of genetic alterations such as somatic mutations and copy number variations. This approach helps elucidate the molecular mechanisms underlying tumorigenesis and provides a basis for developing targeted therapeutic strategies [15]. Analysis of single nucleotide variants (SNVs) revealed a total of 902 SNV sites in this cell line, which were predominantly distributed in intronic regions, CDS regions, and missense SNP regions (Fig. 3A). Somatic InDel analysis revealed 197 insertion or deletion mutations in the genome located across various functional regions (Fig. 3B). On the basis of somatic nonsynonymous mutations (including SNVs and InDels) identified in the patient’s tumor tissue, the tumor mutation burden (TMB) was calculated to assess potential sensitivity to immune checkpoint inhibitors. The TMB value of CBC3T-3 cells was 3.2903, which falls into the low mutation burden category, suggesting that patients may not respond favorably to immune checkpoint inhibitor therapy (Fig. 3C). Single-sample clonal structure analysis revealed significant intratumor heterogeneity (Fig. 3D, Table S1). Furthermore, our set of somatic mutations was compared with a list of known driver genes from the Cancer Gene Census database (sanger.ac.uk), leading to the identification of key driver genes in this tumor sample (Table 2).

**Table 2:** Tumor driver gene of CBC3T-3 cell

Gene	Chrom	Position	Ref	Alt	Classification	AAChange
*PPFIA4*	1	203,015,444	G	T	Missense_Mutation	p.M358I
*NRAS*	1	115,256,529	T	A	Missense_Mutation	p.Q61L
*PBRM1*	3	52,668,805	C	T	Missense_Mutation	p.E372K; p.E358K; p.E390K; p.E393K
*TP53*	17	7,578,269	G	A	Missense_Mutation	p.L62F; p.L35F; p.L155F; p.L194F;
*ELF4*	X	129,203,494	G	C	Missense_Mutation	p.S323C
*PLEC*	8	145,004,325	C	A	Nonsense_Mutation	p.E853X; p.E845X; p.E1004X; p.E835X; p.E867X; p.E871X; p.E894X
*BIRC6*	2	32,770,834	G	C	Missense_Mutation	p.L4238F; p.L4239F
*ARID1B*	6	157,100,301	C	A	Nonsense_Mutation	p.S496X
*NOTCH2*	1	120,471,614	G	A	Missense_Mutation	p.R1293C
*ERCC2*	19	45,867,002	T	C	Missense_Mutation	p.R349G; p.R373G
*EP300*	22	41,572,503	G	T	Nonsense_Mutation	p.E1652X; p.E1678X

*Chrom* chromosome, *Ref* reference base, *Alt* alternative base, *AA change* information on amino acid changes

### Growth characteristics and in vivo tumorigenicity of CBC3T-3 cells

**Fig. 4:**
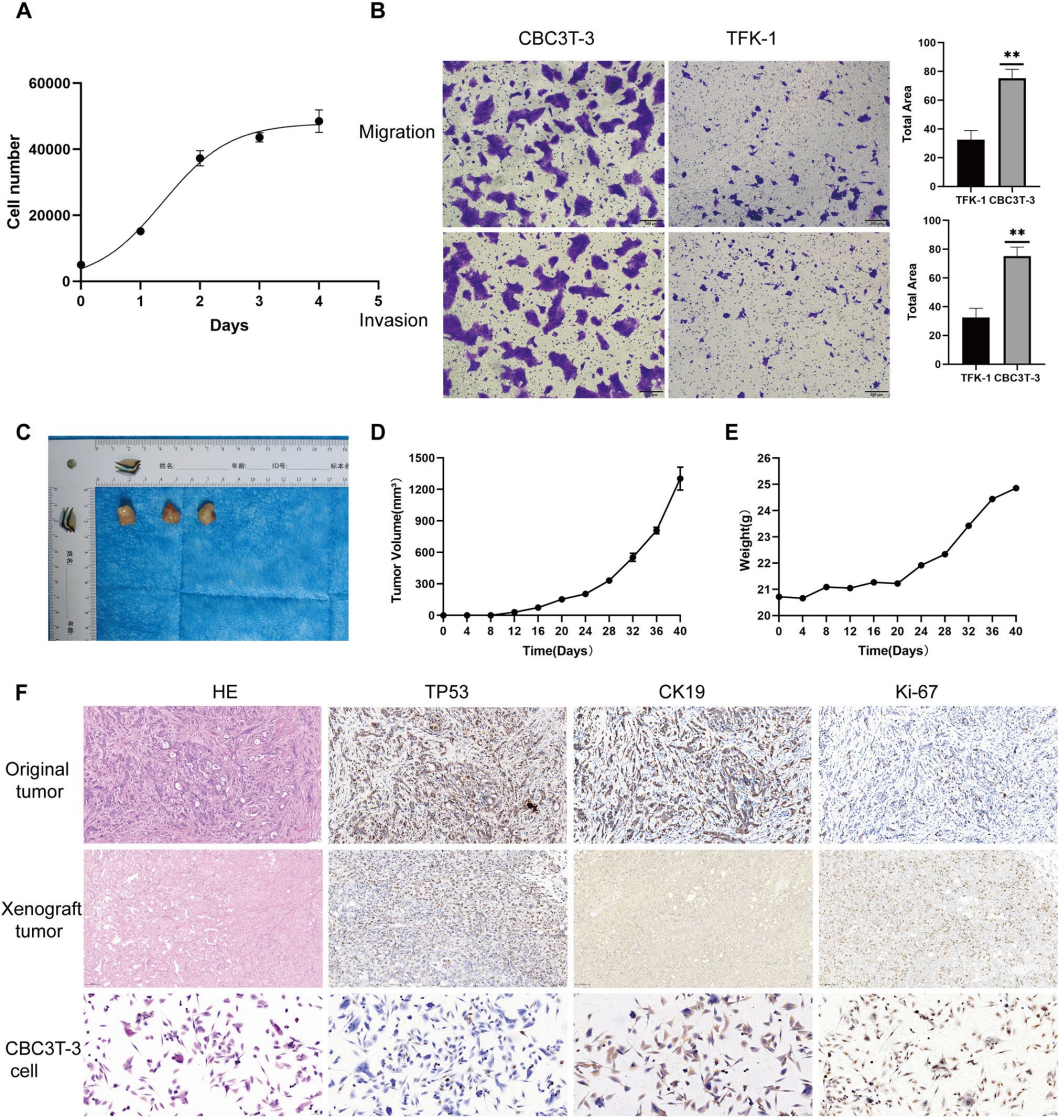
Growth characteristics and in vivo tumorigenicity analysis of CBC3T-3 cells.** A** Growth curve of CBC3T-3 cells. The experiment was performed in triplicate with three technical replicates per condition (n = 3). **B** Representative images and quantitative results of invasion and migration assays in CBC3T-3 and TFK-1 cells (scale bar: 200 μm). **C** Macroscopic view of xenograft tumors derived from CBC3T-3 cells. **D** Growth curve of subcutaneous xenograft tumors in mice. **(E)** Body weight changes in tumor-bearing mice. **F** H&E staining and immunohistochemical results of the original tumor tissue, xenograft tumor tissue and CBC3T-3 cells. Scale bar, 50 μm

On the basis of the growth curve of CBC3T-3 cells, the doubling time (DT) was calculated via the formula DT = (t—t₀) × log (2)/log (N/N₀) and was determined to be approximately 29 h, indicating a relatively rapid proliferation rate (Fig. 4A). Compared with TFK-1 cells, CBC3T-3 cells exhibited significantly greater migration and invasion capabilities (Fig. 4B). After CBC3T-3 cells were inoculated into NOD-SCID mice, palpable tumor nodules formed in all the mice (3/3) within 4 weeks, with no significant decrease in body weight observed, demonstrating strong in vivo tumorigenicity and suitability for further tumor xenograft studies (Fig. 4C–E). The pathological features of the xenograft tumors as well as the CBC3T-3 cells were consistent with those of the patient’s primary tumor. H&E staining revealed that the tumor cells were arranged in sheets or nests, with a large and pleomorphic morphology, eosinophilic cytoplasm, and focal areas of necrosis. Immunohistochemical analysis confirmed the expression of CK19 ( +) and TP53 ( +) in tumor cells, and the Ki-67 labeling index was approximately 20–30% (Fig. 4F).

### Analysis of drug sensitivity in CBC3T-3 cells with TP53 missense mutations

**Fig. 5:**
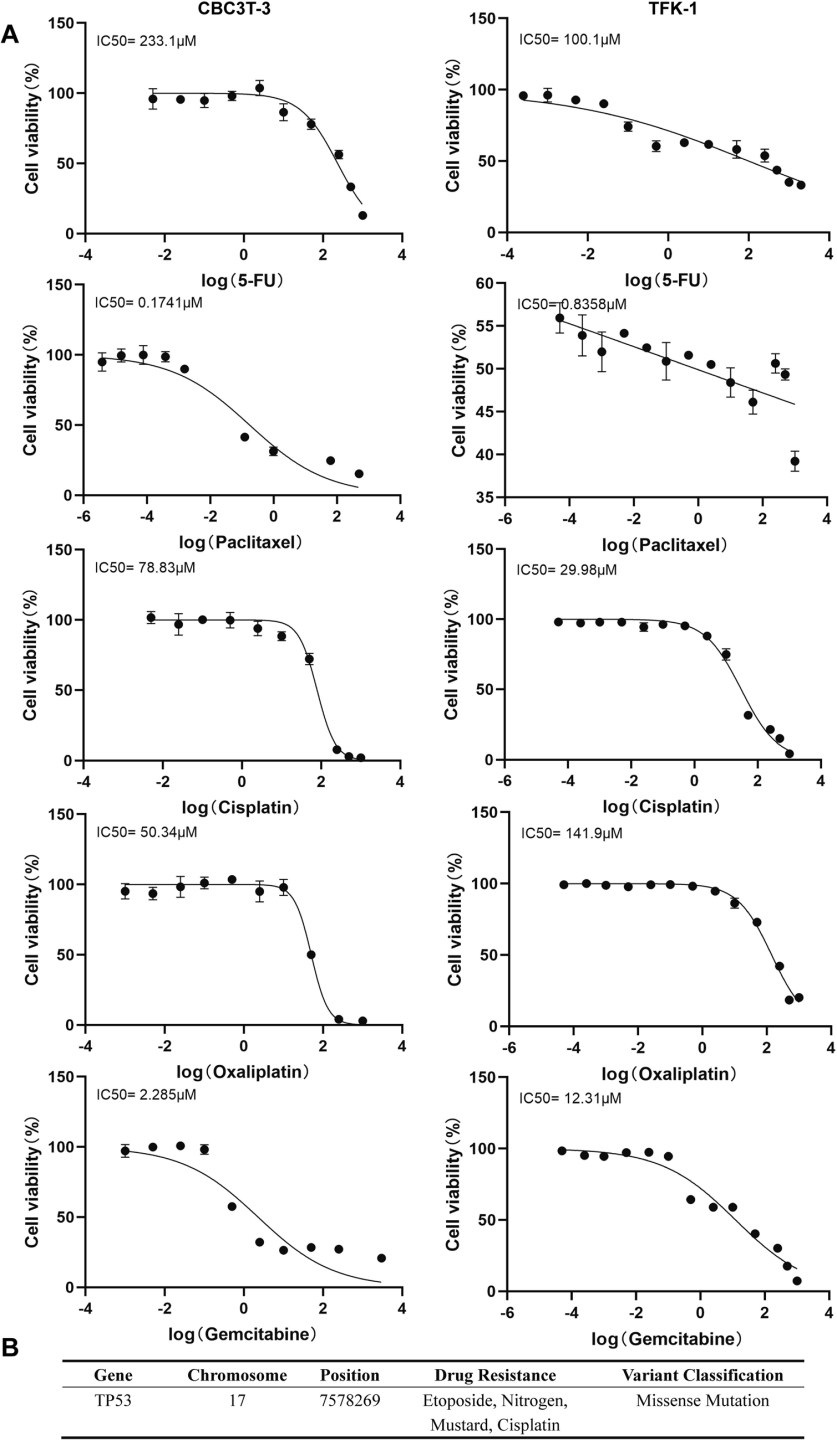
Drug sensitivity analysis of CBC3T-3 cells. **A** Cell viability and IC_50_ values of CBC3T-3 and TFK-1 cells after 48 h of treatment with different concentrations of chemotherapeutic agents (5-FU, gemcitabine, paclitaxel, oxaliplatin, and cisplatin). **B** Screening results of drug resistance mutations via the NovoDR platform

Evaluating the sensitivity of CBC3T-3 cells to various chemotherapeutic agents commonly used in CCA may provide insights for personalized clinical treatment. The establishment of drug-sensitive or drug-resistant cell models could further advance the development of precision therapy for CCA. Compared with TFK-1, CBC3T-3 was shown to be more sensitive to paclitaxel, oxaliplatin and gemcitabine (left-shifted dose‒response curves) and less sensitive to 5-FU and cisplatin (right-shifted dose‒response curves), as shown in Fig. 5A. A comparison of the somatic mutations identified through WES with the NovoDR drug resistance gene database revealed that *TP53* mutations were associated with resistance to agents such as etoposide, nitrogen mustard, and cisplatin (Fig. 5B). These results suggest that multiple *TP53* missense mutations present in CBC3T-3 may contribute to the patient’s insensitivity to cisplatin treatment.

## Discussion

dCCA is a malignant tumor originating from the distal bile duct epithelium. Owing to factors such as delayed diagnosis, high invasiveness, and poor prognosis, its clinical management poses significant challenges [16]. Preclinical tumor models play a vital role in investigating tumor mechanisms and developing novel therapeutics, providing a critical platform for shortening drug development cycles, reducing costs, and advancing personalized treatment [17, 18]. Since the first CCA cell line was established in 1971, over 80 cell lines have been developed worldwide, most of which retain critical biological features of primary tumors [19]. Although China is a region with a high incidence of CCA, cell lines derived from Chinese patients remain relatively scarce [9, 10, 20]. Therefore, establishing CCA cell lines from local patients is crucial for elucidating region-specific pathogenesis and developing targeted therapeutic strategies.

In the present work, we successfully established a new dCCA cell line, CBC3T-3, from the primary tumor tissue of a Chinese male patient (age 54). STR profiling confirmed its genetic uniqueness, supporting CBC3T-3 as a new dCCA model. Karyotype analysis revealed chromosomal instability characterized by a near-triploid karyotype (modal chromosome number 67) with complex numerical and structural abnormalities, which is consistent with the typical cytogenetic features of cancer cells [21]. Morphologically, the cells retained junctional structures of cholangiocytic origin while exhibiting ultrastructural alterations associated with proliferation, migration, and metabolic stress. The cell line demonstrates stable growth in vitro and is capable of forming xenograft tumors in vivo, largely retaining the growth characteristics and histology of the primary tumor. Thus, CBC3T-3 represents a valuable tool for dCCA research with significant potential for scientific applications.

In this study, the cell line was found to harbor numerous somatic mutations, which serve as critical drivers of tumor initiation and progression. Understanding their profiles is essential for advancing cancer prevention and treatment strategies [22]. WES identified several genes harboring multiple somatic amino acid substitutions in CBC3T-3 cells, including *TP53*, *PBRM1*, *ERCC2, BIRC6*, *PLEC* and *EP300.* In CBC3T-3 cells, a mutation was identified at chromosome 17, position 7,578,269 in TP53, resulting in four amino acid substitutions (p.L35F, p.L62F, p.L155F, p.L194F). *TP53* is among the most frequently mutated genes in CCA [23]. Additionally, multiple missense mutations were also found in *PBRM1* (p.E358K, p.E372K, p.E390K), *BIRC6* (p.L4238F, p.L4239F) and *ERCC2* (p.R349G, p.R373G). Furthermore, nonsense mutations were clustered in *PLEC* (p.E853X, p.E845X, p.E1004X, p.E835X, p.E867X, p.E871X, p.E894X), *ARID1B* (p.S496X), and *EP300* (p.E1652X, p.E1678X). *NRAS*, a member of the *RAS* family, can undergo mutations leading to constitutive activation of the protein, thereby driving abnormal cell proliferation and tumorigenesis. The *NRAS* p.Q61L mutation—where glutamine (Q) at position 61 is replaced by leucine (L)—represents the second most common driver event in melanoma, although its role in other cancers remains less defined [24]. Our findings suggest that concurrent *NRAS* and *TP53* mutations contribute to the robust proliferative capacity and genomic instability of CBC3T-3 cells. Furthermore, *PBRM1* and *ARID1B* are core components of the SWI/SNF chromatin remodeling complex, which acts as a tumor suppressor. Dysfunction of this complex due to mutations has been implicated in the pathogenesis of multiple cancer types [25, 26]. This multi-hit pattern within a single gene underscores the genomic complexity of this dCCA model.

*TP53* is a well-known tumor suppressor gene, involved in processes such as cell cycle regulation, DNA damage repair, cell apoptosis, cell senescence, and cell metabolism [27]. *TP53* mutation is one of the most frequent genetic alterations in extrahepatic CCA [28, 29]. *TP53* mutation not only promotes tumor development and progression but is also strongly associated with poor prognosis in CCA patients [23, 30–32].In a pivotal 2017 study, integrated multi-omics analysis of 489 CCA samples led to the classification of the disease into four distinct subgroups with unique molecular profiles. Among these, Cluster 1 and 2 were characterized by prominent *TP53* mutations and *ERBB2* amplifications, and primarily consisted of extrahepatic CCA [28, 33]. Currently, more than 2,000 *TP53* mutations have been discovered, with over 80% being missense mutations predominantly localized in the DNA-binding domain of the *TP53* protein [34]. The common *TP53* hotspot mutations account for approximately 30% of all mutations, while the functions of the remaining mutations remain difficult to elucidate. It is hypothesized that *TP53* mutations can drive tumorigenesis through three mechanisms: loss-of-function, gain-of-function, and dominant negative effects [35–39]. Analysis of the Cancer Cell Line Encyclopedia database revealed that the extrahepatic CCA cell line TFK‑1 harbors a *TP53* nonsense mutation (p. W91Ter), which results in a truncated and functionally inactive *TP53* protein. In CBC3T-3 cells, all four *TP53* mutations (p.L35F, p.L62F, p.L155F and p.L194F) involve the substitution of leucine with phenylalanine, a non-conservative change likely to disrupt local protein structure [40, 41]. In the three-dimensional crystal structure of the *TP53* protein, p.L155F resides in the β-sandwich core critical for structural stability, and p.L194F is located on the DNA-contact surface. These structural disruptions, particularly at the DNA-contact surface, are predicted to severely impair *TP53*’s transcriptional activity. Given that functional *TP53* is central to mediating apoptosis in response to DNA-damaging agents, such mutations are strongly linked to chemotherapy resistance.

Consistent with this mechanistic link, our drug sensitivity analysis provided direct experimental evidence. Gemcitabine combined with cisplatin is the standard first-line chemotherapy regimen for advanced CCA [42]. Drug sensitivity analysis revealed that, compared with TFK-1 cells, CBC3T-3 cells exhibited greater sensitivity to gemcitabine but a poorer response to cisplatin. Screening for resistance mutations via the NovoDR platform suggested that *TP53* mutations are strongly associated with resistance to multiple agents, including cisplatin. Given the presence of *TP53* mutations in this cell line, we speculate that these alterations may be key factors contributing to cisplatin resistance. Multiple studies have established a correlation between *TP53* mutations and cisplatin resistance in cancer patients [43, 44] Previous studies have confirmed that *TP53* mediates apoptosis following DNA damage and that its mutation can disrupt this apoptotic program, leading to resistance to cisplatin in tumor cells [45–47]. In addition to gemcitabine, CBC3T-3 cells also demonstrated high sensitivity to paclitaxel and oxaliplatin. Although *TP53* mutations are generally associated with poor prognosis and chemotherapy resistance, their impact on specific therapeutic agents is highly dependent on the mutation subtype and the broader cellular genetic background. Our findings demonstrate that CBC3T-3 cells remain sensitive to gemcitabine and paclitaxel, suggesting that their particular *TP53* missense variants may bypass canonical resistance mechanisms associated with these drugs. The overall genetic landscape—particularly co‑occurring mutations in genes such as *NRAS* and chromatin remodelers—plays a key role in shaping drug responsiveness in *TP53*‑mutant tumors. It highlights that *TP53* mutation cannot be simplistically viewed as a monolithic predictor of resistance but must be evaluated within its specific mutational spectrum and co-mutation landscape [29]. This further emphasizes the indispensable value of personalized drug testing using patient-derived models for tailoring effective treatment strategies.

In summary, this study successfully established and characterized a novel cell line derived from a Chinese patient with dCCA. The *TP53* missense mutation identified in this cell line is closely associated with a cisplatin-resistant phenotype. As a valuable preclinical model, this cell line not only provides a useful tool for in-depth investigation into the pathogenesis of dCCA but also offers a significant foundation for developing personalized therapeutic strategies on the basis of patient-specific models.

## Supplementary Information

Below is the link to the electronic supplementary material.

Supplementary file1 (DOCX 44 KB)

Supplementary file2 (PDF 79 KB)

## Data Availability

The datasets used and analyzed during the current study are available from the corresponding author upon reasonable request.

## Abbreviations

CCA: Cholangiocarcinoma
CNVs: Copy number variations
dCCA: Distal cholangiocarcinoma
HE: Hematoxylin–eosin
InDels: Insertions and deletions
mar: Marker chromosomes
OD: Optical density
SNPs: Single nucleotide polymorphisms
SNVs: Single nucleotide variants
TMB: Tumor mutational burden
WES: Whole exome sequencing
