# Supplementary material for: CBC3T-3: a novel patient-derived cisplatin-resistant distal cholangiocarcinoma cell line harboring multiple TP53 missense mutations
Source: Hum Cell. 2026 Mar 31;39(4):56. doi: 10.1007/s13577-026-01369-1 (PMC13038642; doi:10.1007/s13577-026-01369-1)
Supplement: Supplementary file 1 — Supplementary file1 (DOCX 44 KB) [file 13577_2026_1369_MOESM1_ESM.docx]

Table S1. Pyclone cluster of CBC3T-3 cell

| Gene | Chromosome | Mutation | Cluster | Cellular prevalence |
| --- | --- | --- | --- | --- |
| *DNAH2* | 17 | 7673692 | 1 | 0.998937796 |
| *TP53* | 17 | 7578269 | 1 | 0.998935672 |
| *BCL6B* | 17 | 6929869 | 1 | 0.998898332 |
| *AGRN* | 1 | 979551 | 1 | 0.998881981 |
| *BMP4* | 14 | 54417379 | 1 | 0.998879706 |
| *NEGR1* | 1 | 72058634 | 1 | 0.998831906 |
| *ABCC9* | 12 | 22047082 | 1 | 0.99882969 |
| *RBM24* | 6 | 17292285 | 1 | 0.99878479 |
| *SYNE1* | 6 | 152763328 | 1 | 0.998677874 |
| *KRT81* | 12 | 52680929 | 1 | 0.998607178 |
| *ARID1B* | 6 | 157100301 | 1 | 0.998436174 |
| *TSPAN11* | 12 | 31135581 | 1 | 0.998290327 |
| *EFNA1* | 1 | 155106245 | 1 | 0.853898265 |
| *TSHB* | 1 | 115576791 | 1 | 0.838583701 |
| *DESI2* | 1 | 244869075 | 1 | 0.823245845 |
| *TRIM2* | 4 | 154216461 | 1 | 0.817566174 |
| *UTP18* | 17 | 49353279 | 1 | 0.815418729 |
| *ADGRB3* | 6 | 70049305 | 1 | 0.809697204 |
| *DPYSL3* | 5 | 146775168 | 1 | 0.800180978 |
| *LRPPRC* | 2 | 44172502 | 1 | 0.794740587 |
| *PTPRN2* | 7 | 157449142 | 1 | 0.793585341 |
| *NRAS* | 1 | 115256529 | 1 | 0.789149227 |
| *TTN* | 2 | 179439710 | 1 | 0.787507862 |
| *GNAI3* | 1 | 110128897 | 1 | 0.787381095 |
| *SOCS7* | 17 | 36552231 | 1 | 0.78610403 |
| *MEOX1* | 17 | 41719159 | 1 | 0.780084843 |
| *HADH* | 4 | 108944664 | 1 | 0.772275229 |
| *MZB1* | 5 | 138723214 | 1 | 0.770067932 |
| *SEMA4F* | 2 | 74900900 | 1 | 0.763819674 |
| *MTRR* | 5 | 7870051 | 1 | 0.759039909 |
| *PROX1* | 1 | 214170906 | 1 | 0.755971369 |
| *TCFL5* | 20 | 61477445 | 1 | 0.754455413 |
| *BARD1* | 2 | 215645363 | 1 | 0.750837368 |
| *ZNF479* | 7 | 57188172 | 1 | 0.750211408 |
| *MCUB* | 4 | 110605805 | 1 | 0.747379673 |
| *TRPM4* | 19 | 49703939 | 1 | 0.746088565 |
| *ENSA* | 1 | 150595134 | 1 | 0.744444477 |
| *GABRA5* | 15 | 27193431 | 1 | 0.739963264 |
| *RAB11FIP1* | 8 | 37720294 | 9 | 0.735842697 |
| *COL20A1* | 20 | 61945141 | 4 | 0.734106824 |
| *RGS7BP* | 5 | 63905375 | 1 | 0.733405095 |
| *NEIL2* | 8 | 11643814 | 1 | 0.728693683 |
| *FSTL5* | 4 | 162380432 | 1 | 0.727865152 |
| *FCRL4* | 1 | 157556013 | 1 | 0.727785644 |
| *JPH2* | 20 | 42788755 | 1 | 0.724193259 |
| *TOGARAM2* | 2 | 29240153 | 23 | 0.722046896 |
| *NR3C2* | 4 | 149357283 | 1 | 0.721304525 |
| *MAP3K7CL* | 21 | 30481679 | 31 | 0.720113457 |
| *CROCC2* | 2 | 241859436 | 13 | 0.719811324 |
| *FGF21* | 19 | 49259531 | 1 | 0.719425275 |
| *VAV3* | 1 | 108298058 | 1 | 0.715277559 |
| *TICRR* | 15 | 90138780 | 1 | 0.712912585 |
| *SETSIP* | 1 | 92540720 | 16 | 0.712496929 |
| *ARHGAP23* | 17 | 36634078 | 1 | 0.709896325 |
| *CFAP69* | 7 | 89929352 | 23 | 0.70940218 |
| *ZGRF1* | 4 | 113502932 | 1 | 0.703993001 |
| *FBN3* | 19 | 8174531 | 8 | 0.698514637 |
| *STEAP2* | 7 | 89859258 | 1 | 0.692434728 |
| *TUBAL3* | 10 | 5437319 | 8 | 0.690019705 |
| *CFAP100* | 3 | 126137503 | 8 | 0.688334652 |
| *CRYBG3* | 3 | 97596799 | 1 | 0.683348527 |
| *ZNF608* | 5 | 124079824 | 1 | 0.682777504 |
| *PHKB* | 16 | 47732393 | 8 | 0.682634278 |
| *FAT2* | 5 | 150925341 | 1 | 0.682225056 |
| *DEFB126* | 20 | 126121 | 1 | 0.680569517 |
| *ANKRD55* | 5 | 55398372 | 1 | 0.673909662 |
| *CPNE7* | 16 | 89651199 | 8 | 0.672238552 |
| *MOV10L1* | 22 | 50588128 | 1 | 0.669141143 |
| *PBRM1* | 3 | 52668805 | 8 | 0.666215115 |
| *TMEM43* | 3 | 14166665 | 8 | 0.660225222 |
| *CKAP5* | 11 | 46772950 | 8 | 0.655851607 |
| *H1-3* | 6 | 26234659 | 3 | 0.647460142 |
| *PIGR* | 1 | 207108844 | 3 | 0.647228953 |
| *UNC13A* | 19 | 17750253 | 8 | 0.645909769 |
| *ANO8* | 19 | 17435976 | 8 | 0.643602504 |
| *GPR158* | 10 | 25464647 | 8 | 0.642844834 |
| *OR10H1* | 19 | 15918638 | 8 | 0.638552145 |
| *SEMA3A* | 7 | 83634741 | 1 | 0.634343871 |
| *TSGA10* | 2 | 99651824 | 3 | 0.629036685 |
| *ADAM23* | 2 | 207435478 | 3 | 0.619784456 |
| *BASP1* | 5 | 17275658 | 32 | 0.616757002 |
| *ZFHX4* | 8 | 77776808 | 3 | 0.608607953 |
| *GABRP* | 5 | 170232841 | 3 | 0.598742199 |
| *OTOG* | 11 | 17634219 | 8 | 0.597852585 |
| *FAM71E1* | 19 | 50979524 | 8 | 0.594456769 |
| *ADARB1* | 21 | 46602620 | 8 | 0.589222432 |
| *GPD1L* | 3 | 32181861 | 8 | 0.585718693 |
| *EIF5AL1* | 10 | 81272742 | 3 | 0.584240908 |
| *UNC13A* | 19 | 17767029 | 34 | 0.579483172 |
| *STXBP1* | 9 | 130453203 | 12 | 0.577965932 |
| *CHST5* | 16 | 75563457 | 28 | 0.576627683 |
| *IQCH* | 15 | 67687881 | 3 | 0.575682437 |
| *OR4C16* | 11 | 55339950 | 8 | 0.575618551 |
| *NCKAP1* | 2 | 183847563 | 8 | 0.575378788 |
| *CACNA2D3* | 3 | 54905616 | 8 | 0.574012934 |
| *RUNX2* | 6 | 45480132 | 3 | 0.567283833 |
| *MEFV* | 16 | 3304695 | 24 | 0.566103797 |
| *ZFHX4* | 8 | 77765901 | 7 | 0.565312837 |
| *OR5F1* | 11 | 55761859 | 8 | 0.56078785 |
| *ABCB11* | 2 | 169820730 | 8 | 0.560719075 |
| *NOXA1* | 9 | 140323343 | 22 | 0.559674018 |
| *OR5K3* | 3 | 98109949 | 30 | 0.555528699 |
| *RALGAPA2* | 20 | 20591988 | 33 | 0.525669081 |
| *CLPP* | 19 | 6366358 | 17 | 0.524188114 |
| *UPK3A* | 22 | 45683271 | 2 | 0.520564977 |
| *KCNMA1* | 10 | 78647205 | 3 | 0.516428923 |
| *ADH1B* | 4 | 100235158 | 3 | 0.511586293 |
| *CDV3* | 3 | 133292966 | 26 | 0.495932946 |
| *PRKAR1B* | 7 | 635833 | 18 | 0.493241824 |
| *PPFIA4* | 1 | 203015444 | 15 | 0.487278311 |
| *NR2F1* | 5 | 92923758 | 3 | 0.45165041 |
| *EP300* | 22 | 41572503 | 20 | 0.445464852 |
| *FREM1* | 9 | 14808125 | 3 | 0.422912304 |
| *ZNF788P* | 19 | 12222627 | 3 | 0.420110492 |
| *ANKRD29* | 18 | 21229114 | 3 | 0.416333491 |
| *NLRP9* | 19 | 56223853 | 3 | 0.415318424 |
| *NOMO2* | 16 | 18532106 | 3 | 0.396638477 |
| *TMPRSS4* | 11 | 117947831 | 21 | 0.38689565 |
| *LRRC23* | 12 | 7023131 | 29 | 0.322769491 |
| *KIF13B* | 8 | 28988104 | 14 | 0.294656789 |
| *SBNO2* | 19 | 1127721 | 11 | 0.271657351 |
| *CCDC50* | 3 | 191075906 | 25 | 0.271609165 |
| *DLG2* | 11 | 84027902 | 19 | 0.263046774 |
| *MFSD4A* | 1 | 205538348 | 5 | 0.254727735 |
| *MFSD4A* | 1 | 205538351 | 10 | 0.254554224 |
| *GATD1* | 11 | 767447 | 6 | 0.253420296 |
| *HNRNPA2B1* | 7 | 26240363 | 27 | 0.23327488 |
| *CD160* | 1 | 145696299 | 0 | 0.224013549 |
| *PDILT* | 16 | 20380957 | 0 | 0.2224264 |
| *UBE2D1* | 10 | 60094785 | 0 | 0.216346827 |
| *TTN* | 2 | 179437150 | 0 | 0.21610522 |
| *ATP10A* | 15 | 25924998 | 0 | 0.216072188 |
| *MUC12* | 7 | 100646629 | 0 | 0.204636499 |
| *SCAF4* | 21 | 33073375 | 0 | 0.204362391 |
| *ADAMTS18* | 16 | 77401445 | 0 | 0.199370138 |
| *SOX6* | 11 | 15994508 | 0 | 0.195007833 |
| *RBFOX1* | 16 | 6704650 | 0 | 0.184921752 |
| *GDF7* | 2 | 20871196 | 0 | 0.178221002 |
| *AKAP13* | 15 | 86028970 | 0 | 0.177735358 |
| *FZD10* | 12 | 130648781 | 0 | 0.176533308 |
| *NPR1* | 1 | 153665901 | 0 | 0.166788044 |
| *PSAT1* | 9 | 980943896 | 0 | 0.16672177 |
| *JAK1* | 1 | 65301124 | 0 | 0.16529016 |
| *MUC12* | 7 | 100646637 | 0 | 0.164920563 |
| *TMEM135* | 11 | 87013337 | 0 | 0.16420263 |
| *RBM4* | 11 | 66413612 | 0 | 0.159249305 |
| *VPS9D1* | 16 | 89774201 | 0 | 0.155866332 |
| *PLXND1* | 3 | 129324445 | 0 | 0.153712845 |
| *OR52E8* | 11 | 5878176 | 0 | 0.153662927 |
| *TNK1* | 17 | 7287478 | 0 | 0.152289296 |
| *MEGF10* | 5 | 126754851 | 0 | 0.150910805 |
| *UBXN1* | 11 | 62445263 | 0 | 0.146878827 |
| *BICC1* | 10 | 60549130 | 0 | 0.146226716 |
| *MYOM2* | 8 | 2092822 | 0 | 0.145920498 |
| *ERCC2* | 19 | 45867002 | 0 | 0.14555472 |
| *FPR1* | 19 | 52249584 | 0 | 0.144243617 |
| *SNPH* | 20 | 1276896 | 0 | 0.143974624 |
| *COL28A1* | 7 | 7516754 | 0 | 0.142178431 |
| *RASGRF1* | 15 | 79320174 | 0 | 0.140476828 |
| *PTGER1* | 19 | 14584426 | 0 | 0.139979331 |
| *MUC12* | 7 | 100634444 | 0 | 0.139961017 |
| *CNOT4* | 7 | 135080584 | 0 | 0.139582238 |
| *COL21A1* | 6 | 56032961 | 0 | 0.138709818 |
| *RYR2* | 1 | 237586394 | 0 | 0.13869246 |
| *MUC12* | 7 | 100634440 | 0 | 0.138545769 |
| *STX16* | 20 | 57242582 | 0 | 0.138360145 |
| *KPRP* | 1 | 152732295 | 0 | 0.138287997 |
| *AP3S1* | 5 | 115249078 | 0 | 0.138185664 |
| *BIRC6* | 2 | 32770834 | 0 | 0.13802152 |
| *CLCNKA* | 1 | 16360206 | 0 | 0.137277079 |
| *HMCN1* | 1 | 186056587 | 0 | 0.137014884 |
| *PLEC* | 8 | 145004325 | 0 | 0.136745334 |
| *SLC30A10* | 1 | 220088999 | 0 | 0.136645331 |
| *CACNA1E* | 1 | 181764049 | 0 | 0.136599752 |
| *PPP2R2B* | 5 | 146257586 | 0 | 0.136574919 |
| *NKX6-3* | 8 | 41507818 | 0 | 0.135681641 |
| *NR1D1* | 17 | 38253017 | 0 | 0.135629153 |
| *OR10G4* | 11 | 123886389 | 0 | 0.135564286 |
| *ADAMTS2* | 5 | 178552091 | 0 | 0.135455218 |
| *FPR1* | 19 | 52249338 | 0 | 0.134913425 |
| *SLC25A46* | 5 | 110074895 | 0 | 0.134457455 |
| *CXCR1* | 2 | 219029293 | 0 | 0.134106238 |
| *PLEKHA8* | 7 | 30157810 | 0 | 0.132780516 |
| *DNAH5* | 5 | 13771079 | 0 | 0.131318572 |
| *POTEC* | 18 | 14542693 | 0 | 0.130156472 |
| *NOTCH2* | 1 | 120471614 | 0 | 0.128226707 |
| *CXCL12* | 10 | 44873589 | 0 | 0.127642421 |
| *ILKAP* | 2 | 239090718 | 0 | 0.124762067 |
| *ADH1A* | 4 | 100203706 | 0 | 0.123939463 |
| *LRRK1* | 15 | 101595345 | 0 | 0.123597728 |
| *OR10G7* | 11 | 123909601 | 0 | 0.120039034 |
| *ABCA12* | 2 | 215818780 | 0 | 0.11977831 |
| *H1-4* | 6 | 26157009 | 0 | 0.119016382 |
| *RHOBTB3* | 5 | 95129595 | 0 | 0.116645993 |
| *GPR32* | 19 | 51274851 | 0 | 0.112573192 |
| *OR10G9* | 11 | 123893827 | 0 | 0.108213602 |
| *KCNK18* | 10 | 118969468 | 0 | 0.093425902 |
