# Supplementary material for: CBC3T-3: a novel patient-derived cisplatin-resistant distal cholangiocarcinoma cell line harboring multiple TP53 missense mutations
Source: Hum Cell. 2026 Mar 31;39(4):56. doi: 10.1007/s13577-026-01369-1 (PMC13038642; doi:10.1007/s13577-026-01369-1)
Supplement: Supplementary file 2 — Supplementary file2 (PDF 79 KB) [file 13577_2026_1369_MOESM2_ESM.pdf]

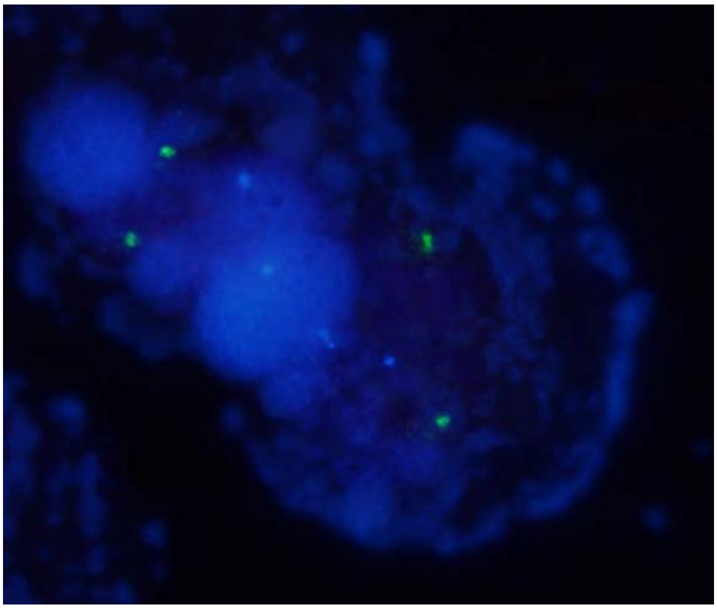

Figure S1. The FISH results of the X and Y chromosome in CBC3T-3 cell

(Chromosome 18: Blue fluorescence signal; X chromosome: Green fluorescence signal; Y chromosome: Orange-red fluorescence signal)
